# Heart-Block

**Published:** 1908-02-01

**Authors:** 


### Clinical Points.

### HEART-BLOCK.

There has been much talk lately about heart-block, and, seeing that the term is one which does not explain itself, it may be worth while saying a few words as to what is meant by it.

It is clear that the term is a bad one, because it might signify so many different things. Mitral stenosis, for example, is an obvious condition of partial blockage inside the heart, and yet mitral stenosis is not heart-block. A clot of blood within the heart, either forming there primarily, or carried there as a large embolus, is another condition which might merit the term; or a blockage in coronary artery might be regarded, with somewhat less correctness, as a heart-block.

The term, however, means none of these. It is arbitrarily restricted to that condition of things in which some of the contractions of the auricle fail to be followed by contractions of the ventricle. It may be that only every alternate contraction of the auricle leads to a contraction of the ventricle; or every third; or it may be that it is only now and then, or, at any rate, with no regularity, that the auricular contractions fail to be followed by contractions of the ventricle. In the first of these cases the ventricular contractions would be precisely half as frequent as those of the auricles; in the second one third as frequent; and in the third case the frequency of the ventricular beats would bear no definite ratio to the frequency of the auricular beats, though the ventricle would contract less frequently than would the auricle.

Precisely similar conditions have been produced experimentally in animals by using a mechanical apparatus such as a clamp to make it more difficult than usual for the auricular contractions to be transmitted to the ventricles. The action of the clamp, or "block," is so exactly like that of the effects of certain pathological lesions in man that the condition, when it arises from disease, has been styled heart-block. The term heart-block is very difficult to replace by any other which is equally succinct, so that, unsatisfactory though it may be from an etymological point of view, it is likely to be retained, and to be given this arbitrary meaning.

### The Methods of Detecting Heart-Block.

The most accurate method of detecting heart-block and investigating it fully is that of taking synchronous tracings from the jugular vein in the neck, and from the radial artery at the wrist. The jugular vein tracing records the contractions of the right auricle, and the radial artery movements record the contractions of the left ventricle. It is unfortunate that we cannot obtain records of the contractions of the left auricle at the same time as those of the left ventricle, or of those of the right ventricle simultaneously with those of the right auricle; we cannot do so, and we must be content with the records obtainable. It is by Dr. Mackenzie in this country and by Professor Wenckebach on the Continent that much of the research work upon the subject has been done. Their labours have been immense, and amongst the definite outcomes of them must be counted a great deal of our knowledge of heart-block.

In the private practice of most of us it is hardly possible to take synchronous tracings from jugular vein and radial artery. Indeed, to do so requires much skill, and a long period of training. Nevertheless, now that we have been told what to look for, we can learn a good deal without actual tracings. It is often possible to see the variations in the jugular vein, and hence to count the venous pulse in the neck in conjunction with the arterial pulse at the wrist; in this way, though there are certain errors that may creep in from lack of delicacy in detecting small pulsations with the eye or finger, a good deal may be learned; and if it is clear that the jugular vein pulsates twice for every once of the radial artery, it is equally clear that the patient's condition is one of heart-block.

Another way in which some clinical (bedside) information on the subject may be obtained is to listen very carefully over the base of the heart, whilst the palm of the hand is at the same time held over the cardiac impulse. It will be possible in some cases to make quite sure that the auricle contracts more often than does the ventricle; the sounds made by the auricle sometimes continuing with regularity, whilst the ventricular sound may be absent either regularly or irregularly, the impulse being found wanting at the same times. It is true that in some cases heart-block may be present without being detected in this way, owing to there being too great an irregularity for the ear to discriminate between the sounds; but the method may be of great value in other cases, and when it is realised what it is one is listening for, it becomes less difficult to detect it than might perhaps be expected.

### The Nature and Origin of Heart-Block.

The nomenclature that has arisen in connection with cardiac phenomena, and the complexity of the graphic records that have been published both in the journals and in book form, have made it a little difficult to understand all the things that have been written upon the subject. We will try and avoid as much of the complexity as possible in an endeavour to make clear what heart-block depends on.

To begin with, we are not going to discuss whether the movements of the heart depend upon the heart-muscle primarily, or upon the nerves supplying that muscle; whether the origin of the cardiac action is myogenic, neurogenic, neuro-myogenic, or myo-neurogenic does not matter greatly to us just now, but we must be clear that there are many different functions concerned in the heart's action. These different functions are:—

1. *Contractility* (*i.e.* ability to contract at all).
2. *Rhythmicity* (*i.e.* ability to contract again and again at more or less regular intervals).
3. *Excitability* (*i.e.* ability to be made to contract as the result of stimulation from without or from within).

4. *Tonicity* (*i.e.* ability to maintain a certain degree of contraction or tone even during diastole).
5. *Conductivity* (*i.e.* ability to transmit the wave of contraction from one part of the heart to another).

It is clear that, theoretically, any one of the above functions might be upset without the others being affected, and that trouble would result in each case. The only two of the above that need be discussed in connection with heart-block are *excitability* and *conductivity*, and for the sake of clearness we shall discuss only the latter, namely conductivity.

### How is the Auricular Contraction Normally Conducted to the Ventricle?

The above question has been studied by physiologists for many years, and there are various theories upon the subject. We shall not discuss all these, but will give only that which is now generally accepted. This entails a description of a structure which all of us have read about, but which many of us have taken for granted without attaching a great deal of importance to it; indeed, one has perhaps been rather inclined to dismiss it from one's thoughts as being little more than an unnecessary refinement in anatomy. This structure is the auriculo-ventricular band of His. It is sometimes spoken of as the auriculo-ventricular *muscle*, but we shall say a word or two on this point presently. It has recently been studied with much care by Keith and Flack in England, and by a Japanese observer named Tawara, and by dint of most careful and laborious dissection it has been shown to be not merely a muscular coupling or link between the lowest part of the auricle and the upper edge of the ventricle, but a most widespreading system of fibres which, gathered together from all over the wall of the auricle, unite to form the band itself at the auriculo-ventricular junction, and then, branching again, ramify all over the ventricular wall, including the musculi papillares. The fibres of this band and its ramifications can be distinguished microscopically from ordinary heart-muscle fibres.

It is a point of special interest to note that the auriculo-ventricular band of His does not hypertrophy when from any cause there is hypertrophy of the general musculature of the heart; this agrees with the view that the band is not muscular, but nervous.

One of the ways in which heart-block may arise is by disease of this auriculo-ventricular band. It is not to be supposed that this is the only possible cause of heart-block. It is conceivable, for example, that the band might be quite healthy, but the excitability of the ventricle so diminished as to require a repeated stimulus, and therefore the ventricle might respond only to some of the auricular contractions; or, again, it is conceivable that the auriculo-ventricular band might be quite healthy, and yet owing to something wrong higher up in the chain, no proper stimuli might be sent off along the band at all. Post-mortem examinations in cases of heart-block, however, have nearly always shown disease of the auriculo-ventricular band whenever this has been carefully sought for. Sometimes a gross lesion such as a gumma has been found replacing the band entirely; sometimes the band has simply degenerated, partly or completely. When the degeneration is partial, stimuli can still pass along it but the conductivity is diminished, so that the ventricle responds with regularity, but only to every second or every third auricular contraction. When the degeneration of the band is complete the ventricle no longer beats in relation to the auricle at all, but in its own innate rhythm, which is always slower than that of the auricle.

### The Relation of Stokes-Adams Disease to Heart-Block.

It is not every day that one sees a case of Stokes-Adams disease; but when looked for the condition is less uncommon than one might think. The main outward feature of the disease is the periodic seizure of the patient by a "fit," which may have any degree of severity from that which resembles petit mal, to much more alarming attacks, in which the patient loses consciousness completely, and may either lie still, as in a syncope (the apoplectiform variety), or struggle in alternating tonic and clonic convulsions (the epileptiform variety). Many of the cases might be taken for epilepsy, but the clinching point in the diagnosis is the pulse rate. At ordinary times this may be, say, 64; and during the "fit" it may be only 32; if the pulsations in the jugular veins can be counted at the same time these may be found to be still 64—in other words, the patient is suffering from a condition of recurrent heart-block, each convulsive seizure being synchronous with the failure of the ventricle to respond to every contraction of the auricle. One premises in these cases that the auriculo-ventricular band of His is not entirely gone, but that it is defective; and that, whereas as a rule it manages to conduct the impulses sufficiently well from auricle to ventricle, at times it fails to do so; at such times the sudden halving of the frequency of the ventricular beats causes an upset in the circulation, including that of the brain, with the result that unconsciousness and epileptiform convulsions ensue.

It will be gathered from what has just been said that it is most important to count the pulse-rate in every case of apparent epilepsy during the seizure, and to compare its frequency with that which is normal for the patient. If the pulse be not counted it is possible to regard as ordinary epilepsy that which is really heart-block.

It seems that of all the functions of the heart which we have enumerated above, those which are most beneficially affected by digitalis are rhythmicity and contractility—or rather the disorders of these. More work is still needed upon the subject, but it appears that digitalis gives little benefit when the function of the heart which is disordered is mainly its conductivity. Further than this, digitalis may be a very dangerous drug when the conductivity is wrong. The drug should not be given in cases of heart-block. It is well known that in a few heart conditions digitalis is a veritable poison; the thing to look out for is evidence of heart-block, and, if any signs of it are found, one must be particularly mindful of the dangers of digitalis in that case.

